# 

**Published:** 1873-04

**Authors:** 


### Contents of April Number.

### ORIGINAL DEPARTMENT.

ART. I.—Clinical Experiences in Private Practice. By Z. C. McElroy, M. D. .... 321
ART. II.—Medical Society of the County of Albany, Semi-Monthly Meetings. Reported by F. C. Curtis, M. D., Sect'y.... 331
ART. III.—Electricity as a Therapeutic Agent. By G. W. Frion, M. D. 337
ART. IV.—Use of Placental Forceps through the Speculum. By H. Seaman, M. D.... 340
ART. V.—Decision in a Suit of Malpractice.... 343

### MISCELLANEOUS.

Death from a Homœopathic Dose of Chloral.... 345

Digestion of Starch in Infancy .... 346

### EDITORIAL DEPARTMENT.

Quackery and Superstition .... 347
Association of American Medical Editors.... 347
The Toner Lectures .... 347
American Medical Association.... 348
Alarm from Rumor of the Prevalence of Child-Bed Fever.... 348

BOOKS REVIEWED:

Diseases of the Ovaries. By T. Spencer Wells .... 349
Clinical Lectures on Diseases Peculiar to Women. By Lombe Atthill, M. D.... 350
Disease of the Rectum, their Diagnosis and Treatment. By Wm. Allingham, F. R., C. S.... 351
The U. S. Pharmacopœia .... 351
A Treatise on the Theory and Practice of Obstetrics. By Wm. H. Byford, A. M., M. D.... 351
Operative Surgery, adapted to the Living and the Dead Subject. By C. F. Maunder .... 352
Illustrations of the Influence of the Mind upon the Body in Health and Disease. By Daniel Hack Tuke, M. D., M. R. C. P.... 353
The Practice of Surgery. By Thomas Bryant, F. R. C. S.. 354
Contributions to Mental Pathology. By I. Ray, M. D..... 355
A Treatise on Apoplexy. By John A. Lidell, A. M., M. D.. 356
Medical and Surgical History of the War of the Rebellion .. 356
The Science and Art of Surgery. By John Eric Erichsen .. 357
First Annual Report of the Supervising Surgeon of the Marine Hospital Service of the United States.... 358

Books and Pamphlets Received.... 359

